# PTP-PEST targets a novel tyrosine site in p120 catenin to control epithelial cell motility and Rho GTPase activity

**DOI:** 10.1242/jcs.120154

**Published:** 2014-02-01

**Authors:** Rosario Espejo, Yowjiun Jeng, Adriana Paulucci-Holthauzen, William Rengifo-Cam, Krysta Honkus, Panos Z. Anastasiadis, Sarita K. Sastry

**Affiliations:** 1 Sealy Center for Cancer Biology and UTMB Comprehensive Cancer Center, University of Texas Medical Branch, Galveston, TX 77555, USA; 2 Department of Biochemistry and Molecular Biology, University of Texas Medical Branch, Galveston, TX 77555, USA; 3 Center for Biomedical Engineering, University of Texas Medical Branch, Galveston, TX 77555, USA; 4 Department of Cancer Biology, Mayo Clinic, Jacksonville, FL 32224, USA

**Keywords:** PTP, Rho GTPase, Cell motility, p120 catenin

## Abstract

Tyrosine phosphorylation is implicated in regulating the adherens junction protein, p120 catenin (p120), however, the mechanisms are not well defined. Here, we show, using substrate trapping, that p120 is a direct target of the protein tyrosine phosphatase, PTP-PEST, in epithelial cells. Stable shRNA knockdown of PTP-PEST in colon carcinoma cells results in an increased cytosolic pool of p120 concomitant with its enhanced tyrosine phosphorylation and decreased association with E-cadherin. Consistent with this, PTP-PEST knockdown cells exhibit increased motility, enhanced Rac1 and decreased RhoA activity on a collagen substrate. Furthermore, p120 localization is enhanced at actin-rich protrusions and lamellipodia and has an increased association with the guanine nucleotide exchange factor, VAV2, and cortactin. Exchange factor activity of VAV2 is enhanced by PTP-PEST knockdown whereas overexpression of a VAV2 C-terminal domain or DH domain mutant blocks cell motility. Analysis of point mutations identified tyrosine 335 in the N-terminal domain of p120 as the site of PTP-PEST dephosphorylation. A Y335F mutant of p120 failed to induce the ‘p120 phenotype’, interact with VAV2, stimulate cell motility or activate Rac1. Together, these data suggest that PTP-PEST affects epithelial cell motility by controlling the distribution and phosphorylation of p120 and its availability to control Rho GTPase activity.

## INTRODUCTION

The armadillo family of cytoplasmic proteins, the catenins, are integral components of adherens junctions in epithelial cells (reviewed by Gumbiner, 2005; Hartsock and Nelson, 2008). The assembly and maintenance of adherens junctions relies on the association of catenins with the E-cadherin cytoplasmic domain to preserve epithelial integrity. Oncogenic tyrosine kinases, such as EGFR and src family kinases, phosphorylate junctional components causing disruption of static cell–cell adhesion. An important consequence of this disruption is enhanced motility and tumor cell invasion. In particular, p120 catenin (p120) plays a crucial role in regulating E-cadherin-mediated cell–cell adhesion versus cell motility (Reynolds and Roczniak-Ferguson, 2004; Anastasiadis, 2007). These phenotypes are highly dependent upon the subcellular distribution of p120. When bound to the E-cadherin juxtamembrane domain, p120 stabilizes E-cadherin on the cell surface, preventing receptor internalization by the endocytic pathway (Davis et al., 2003) to promote E-cadherin homophilic adhesion. In the cytosol, p120 stimulates cell motility (Noren et al., 2000; Anastasiadis et al., 2000; Grosheva et al., 2001; Yap and Kovacs, 2003). The significance of the membrane versus cytosolic distribution of p120 is underscored by studies showing that altered distribution of p120 in human cancers correlated with tumorigenicity, anoikis resistance, poor patient outcome and aggressive metastatic disease (Smalley-Freed et al., 2011; Schackmann et al., 2011; Bellovin et al., 2005). However, the factors controlling p120 subcellular distribution are not well defined.

p120 is a highly tyrosine phosphorylated protein, first identified in v-src-transformed cells (Reynolds et al., 1994). p120 has many isoforms in epithelial cells – the 1A, 2A, 3A and 4A isoforms being predominant (Mo and Reynolds, 1996; Keirsebilck et al., 1998). All p120 isoforms bind to E-cadherin through the central armadillo domains to preserve epithelial adhesion (Reynolds and Roczniak-Ferguson, 2004; Anastasiadis, 2007). However, only the 1A isoform promotes motility (Soto et al., 2008), and overexpression studies attribute this effect to the N-terminal region (Yanagisawa et al., 2008; Aho et al., 2002). It has been proposed that tyrosine phosphorylation of p120 controls its association with E-cadherin as well as its role in cell motility. How tyrosine phosphorylation of p120 controls motility versus E-cadherin association is not clear. Thirty-seven tyrosine residues are present within the primary sequence of the p120 1A isoform and the majority of these sites are within the N-terminal domain. To date, nine tyrosines have been identified as phosphorylation sites in the p120 N-terminal domain (Mariner et al., 2001; Castaño et al., 2007). Some of these sites may be important for signaling to Rho GTPases, but they do not appear to directly affect p120 binding to E-cadherin or cell motility (Castaño et al., 2007; Macpherson et al., 2007). Thus, it is conceivable that additional tyrosine phosphorylation sites may be present either in the N-terminal domain or elsewhere in the protein that control these functions.

It is well established that cytosolic p120 controls cell motility through regulation of Rho GTPase activity (Noren et al., 2000; Anastasiadis et al., 2000; Grosheva et al., 2001). Overexpression of p120 enhances the activity of Rac1 and cdc42 while suppressing activity of RhoA (Noren et al., 2000; Anastasiadis et al., 2000; Grosheva et al., 2001). p120-mediated activation of Rac and cdc42 are proposed to occur through its association with the guanine exchange factor, VAV2 (Noren et al., 2000). Recently, Rac1b was shown to directly bind p120, suggesting an additional level of regulation (Orlichenko et al., 2010). p120 directly regulates RhoA activity by functioning as a Rho-guanine dissociation inhibitor (GDI) to sequester Rho-GDP. Tyrosine phosphorylation of p120 is implicated in this activity. Src-mediated phosphorylation of tyrosines 217 and 228 promotes RhoA-GDP association whereas Fyn-mediated phosphorylation of tyrosine 112 blocks GDI activity (Castaño et al., 2007). p120 associates with several additional cytoplasmic and cytoskeletal proteins including cortactin, p190RhoGAP, kinesin and tyrosine phosphatases (Boguslavsky et al., 2007; Wildenberg et al., 2006; Yanagisawa et al., 2004; Smith et al., 2012; Zondag et al., 2000). A recent study showed that p120 is localized at the leading edge of migrating epithelial cells where it interacts with cortactin to promote actin polymerization (Boguslavsky et al., 2007). An important unanswered question is which of these interactions that are crucial for cell motility are dependent on tyrosine phosphorylation? Additionally, what are the negative regulators of p120 in epithelial cells?

Protein-tyrosine phosphatase non-receptor type 12 (PTP-PEST) is a cytoplasmic tyrosine phosphatase previously shown to regulate fibroblast cell motility and Rho GTPase activity (Angers-Loustau et al., 1999; Garton and Tonks, 1999; Sastry et al., 2002; Sastry et al., 2006). Recent studies have shown that PTP-PEST also controls epithelial cell motility by acting on tyrosine kinases including src, abl, FAK and EGFR (Mathew et al., 2008; Taieb et al., 2008; Zheng et al., 2009; Sun et al., 2011). We recently demonstrated that PTP-PEST is required to suppress colon carcinoma cell motility and maintain adherens junction integrity (Espejo et al., 2010). Using stable shRNA knockdown, we showed that decreased PTP-PEST expression enhanced chemotaxis and haptotaxis of colon carcinoma cells and induced a mesenchymal phenotype. PTP-PEST knockdown also disrupted adherens junction assembly and enhanced Rac1 activity while decreasing activation of RhoA in response to E-cadherin engagement. These findings suggested that PTP-PEST acts on a junctional target to mediate its effects. We show that PTP-PEST controls the subcellular distribution and tyrosine phosphorylation of p120. Silencing of PTP-PEST by RNA interference disrupts the E-cadherin–p120 interaction and enhances the cytosolic pool of p120 to mimic p120 overexpression in epithelial cells. We show that p120 is a direct substrate of PTP-PEST and that tyrosine 335 in the N-terminal domain of p120 is a novel site of dephosphorylation. Cytosolic p120 accumulates in lamellipodia and has an increased association with the guanine exchange factor, VAV2 and the actin-binding protein cortactin in cells depleted of PTP-PEST by shRNA. Collectively, our data support the hypothesis that PTP-PEST inhibits the pro-migratory, cytosolic functions of p120 through dephosphorylation of a novel tyrosine residue. Thus, PTP-PEST controls the phosphorylation and distribution of p120 and its availability to regulate Rho GTPases at the leading edge of migrating epithelial cells.

## RESULTS

### p120 catenin is a substrate of PTP-PEST

**Fig. 1.**
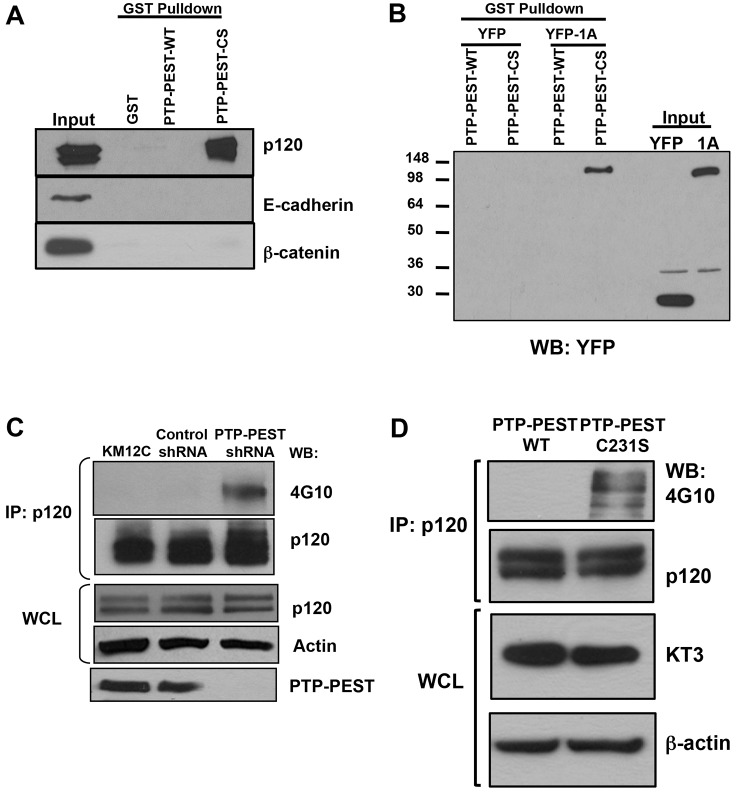
**p120 is a substrate of PTP-PEST in epithelial cells.** (A,B) GST pulldown substrate trapping assay shows (A) endogenous p120 interacts with the GST–C231S trapping mutant of PTP-PEST (PTP-PEST-CS) but not GST–PTP-PEST wild type (PTP-PEST-WT) or GST alone in pervanadate-treated KM12C colon carcinoma cells and (B) ectopic YFP-tagged p120-1A isoform traps with the PTP-PEST-CS fusion protein, but not PTP-PEST-WT, in HEK 293T cells treated with pervanadate. YFP alone does not interact with either fusion protein. (C) Endogenous p120 is hyperphosphorylated on tyrosine in KM12C colon carcinoma cells with stable shRNA knockdown of PTP-PEST. Endogenous p120 was immunoprecipitated and phospho-p120 was detected by western blotting with anti-phosphotyrosine antibody, 4G10. (D) Transient re-expression of KT3-tagged wild-type PTP-PEST cDNA, but not the C231S mutant, in PTP-PEST knockdown cells suppresses enhanced tyrosine phosphorylation of p120. Transfected PTP-PEST was detected with anti-KT3 epitope tag antibody and β-actin is shown as a loading control. WCL, whole cell lysate.

Our previous studies show that PTP-PEST plays a crucial role in controlling epithelial cell motility, adherens junction assembly and Rho GTPase activity in colon carcinoma cells (Espejo et al., 2010). Therefore, we sought to identify targets of PTP-PEST in epithelial cells. Substrate trapping is a method used to specifically identify phosphotyrosine targets of protein tyrosine phosphatases (Flint et al., 1997; Sastry et al., 2006). In this method, a glutathione *S*-transferase (GST) fusion protein encoding a catalytically inactive phosphatase is incubated with cell lysates. Candidate substrates are identified by western blot analysis as positively interacting with the inactive phosphatase, thus ‘trapped’, whereas the wild-type phosphatase shows no interaction. Here, we utilized substrate trapping to determine whether adherens junction proteins were direct targets of PTP-PEST. KM12C colon cancer cells were treated with pervanadate to inactivate all tyrosine phosphatases and GST pulldowns using GST, GST–PTP-PEST-WT or GST–PTP-PEST-CS (catalytically inactive C231S trapping mutant) were analyzed by western blotting for E-cadherin, α-catenin, β-catenin and p120 catenin (p120). Western analysis of GST–C231S pulldown shows that only p120 is a substrate of PTP-PEST (Fig. 1A). No interaction was observed between p120 and GST–PTP-PEST-WT or GST alone. Neither E-cadherin, β-catenin (Fig. 1A) nor α-catenin (data not shown) were detected in the trapping mutant pulldown (Fig. 1A). Similar results were observed with HEK 293T cells and MDCK cells (data not shown). To test whether PTP-PEST trapping mutant recognized exogenous p120, we transiently expressed a cDNA encoding the full-length p120-1A isoform, YFP-tagged p120-1A (YFP–1A) or YFP only in HEK 293T cells. As seen in Fig. 1B, YFP–1A was recognized by the C231S trapping mutant but not by wild-type PTP-PEST. To determine whether PTP-PEST affected tyrosine phosphorylation of p120, we compared phosphorylated p120 levels in KM12C cells with stable shRNA knockdown (KD) of PTP-PEST (KM12C-KD) and KM12C cells expressing non-targeting shRNA (KM12C-NT). In KM12C-KD cells, basal tyrosine phosphorylation of p120 was enhanced compared with KM12C-NT cells (Fig. 1C). Finally, to demonstrate that p120 tyrosine phosphorylation was dependent on PTP-PEST activity, we re-expressed wild-type or C231S PTP-PEST cDNA in KM12C-KD cells and performed immunoprecipitation to examine p120 phosphorylation status. As shown in Fig. 1D, transient expression of KT3-tagged wild-type PTP-PEST suppressed tyrosine phosphorylation of p120 in the knockdown cells. In contrast, expression of the C231S inactive PTP-PEST did not affect p120 tyrosine phosphorylation, indicating that this effect is mediated by PTP-PEST catalytic activity. Taken together these results demonstrate that p120 is a substrate of PTP-PEST.

### PTP-PEST regulates p120-E-cadherin association and shuttling to the cytosol

**Fig. 2.**
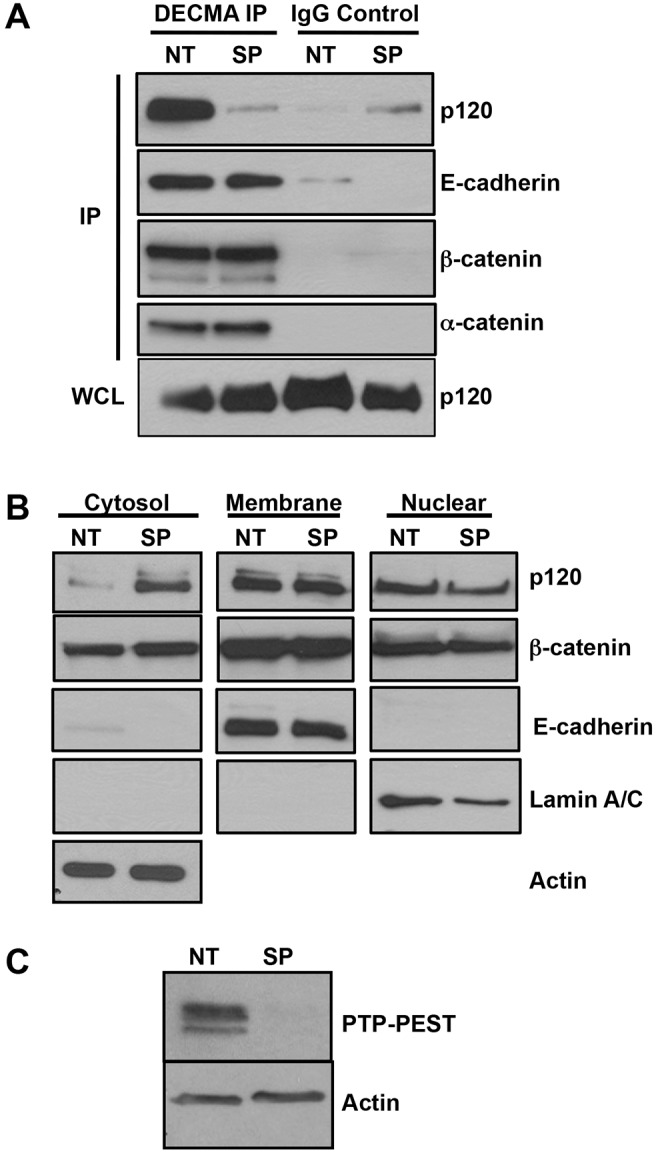
**PTP-PEST regulates p120 subcellular distribution.** (A) PTP-PEST regulates p120 association with E-cadherin. E-cadherin was immunoprecipitated with DECMA-1 antibody from HCT116 cells transfected with a control, non-targeting siRNA (NT) or smart pool PTP-PEST siRNA (SP). Murine IgG was used as a negative control. Immunoprecipitates were analyzed by western blotting for p120 and E-cadherin. Silencing of PTP-PEST selectively disrupts E-cadherin–p120 interaction, but not E-cadherin binding to β-catenin or α-catenin. (B) Silencing PTP-PEST enhances the cytosolic pool of p120. HCT116 cells treated with control (NT) or PTP-PEST smart pool siRNA (SP) were separated into membrane, cytoplasmic or nuclear fractions (as described in Materials and Methods). Western blot analysis shows increased p120 in the cytoplasmic fraction following PTP-PEST knockdown; however, the membrane and nuclear pools of p120 remain unchanged. β-actin, E-cadherin and lamin A/C serve as cytosol, membrane or nuclear markers, respectively. (C) Western blot showing efficient knockdown of PTP-PEST by smart pool siRNA (SP) relative to non-targeting control (NT). β-actin is shown as a loading control.

A key property of p120 is its ability to shuttle between distinct subcellular compartments. When bound to E-cadherin, p120 stabilizes adherens junctions (Davis et al., 2003). In contrast, elevated levels of p120 in the cytosol promote cell motility (Noren et al., 2000; Anastasiadis et al., 2000; Grosheva et al., 2001) and nuclear p120 binds to and inhibits the transcriptional repressor Kaiso (Daniel and Reynolds, 1999). Based on our recent findings that PTP-PEST knockdown disrupts E-cadherin junction assembly (Espejo et al., 2010), we tested whether PTP-PEST affected p120 association with E-cadherin. Immunoprecipitation of cell surface E-cadherin followed by western blotting showed reduced association of p120 with E-cadherin in HCT116 cells transfected with PTP-PEST smart pool siRNA (SP) relative to a non-targeting control (NT; Fig. 2A). This effect was specific to p120 and did not alter the binding of β-catenin or α-catenin to E-cadherin (Fig. 2A). Furthermore, as previously reported, PTP-PEST knockdown did not affect the total protein level of p120 or E-cadherin (Espejo et al., 2010). We next performed subcellular fractionation to determine whether PTP-PEST knockdown affected the subcellular distribution of p120. As seen in Fig. 2B, siRNA knockdown of PTP-PEST in HCT116 cells resulted in increased levels of cytosolic p120, but not membrane-associated or nuclear p120. This effect was specific to p120 because β-catenin showed no change in distribution under the same conditions. β-actin, E-cadherin and lamin A/C are shown as controls for purity of the fractions tested. Fig. 2C shows efficient knockdown of PTP-PEST by the smart pool siRNA. Similar results were obtained in KM12C and MDCK cells (data not shown). Therefore, PTP-PEST regulates the association of p120 with E-cadherin and its shuttling to the cytosol.

### PTP-PEST knockdown mimics p120 overexpression

**Fig. 3.**
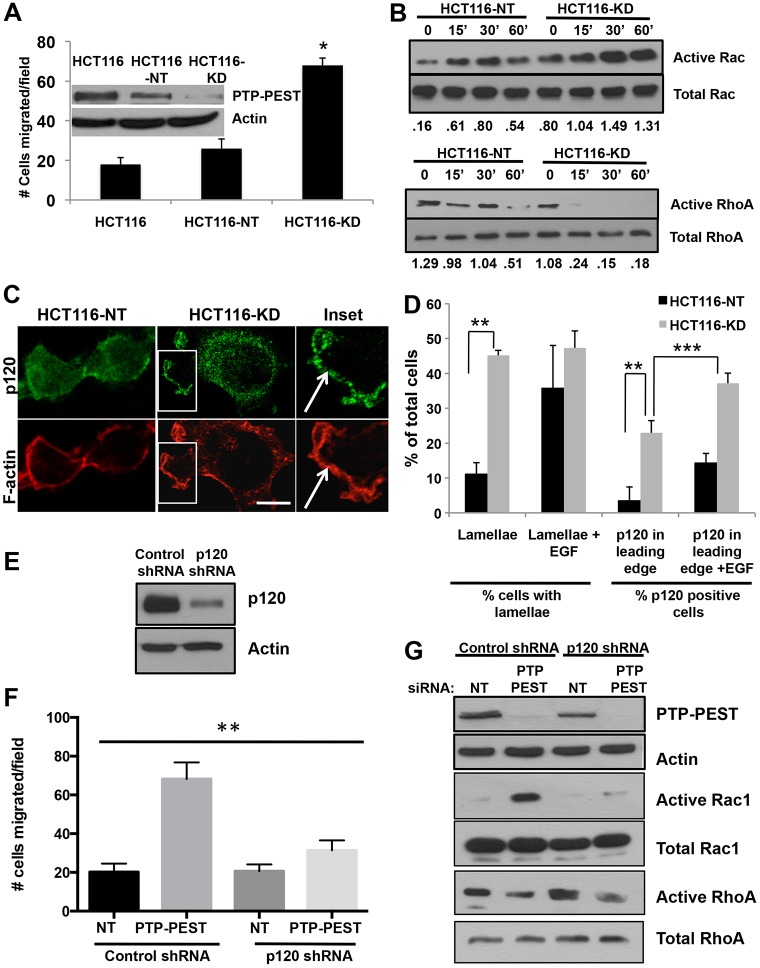
**Stable shRNA silencing of PTP-PEST mimics p120 overexpression effects.** Retroviral infection was used to generate HCT116 cell lines with stable knockdown of PTP-PEST using PTP-PEST shRNA (HCT116-KD) or control, non-targeting shRNA (HCT116-NT) as described in Materials and Methods. (A) PTP-PEST knockdown promotes enhanced haptotaxis to a collagen I substrate. Migration of HCT116-NT or HCT116-KD cells towards collagen I in a Transwell assay was analyzed after 4 hours. *P<0.005, one-way ANOVA. (B) GST–PBD or GST–RBD pulldown assays were used to measure activation of Rac1 and RhoA in HCT116-NT and HCT116-KD cells held in suspension (time = 0) or plated on collagen I-coated plates for the times indicated. Silencing of PTP-PEST enhances Rac1 (top panels) and suppresses RhoA activity (bottom panels). Densitometry was performed on immunoblots using Metamorph software and plotted as a ratio of the band intensity for pulldown divided by intensity of total Rac1 or RhoA present in the cell lysate. Data are representative of three independent experiments. (C) PTP-PEST knockdown enhances lamellipodia and membrane ruffle formation, and p120 localization is enhanced at the leading edge of lamellipodia (see ‘inset’ from the middle panels). HCT116-NT or HCT116-KD cells were plated on collagen-coated (15 µg/ml) coverslips for 90 minutes followed by treatment with EGF (25 ng/ml) for 15 minutes and stained for p120 (green) or F-actin (red). Scale bar: 20 µm. (D) The percentage of cells with lamellipodia and leading-edge staining for p120 were quantified (*n* = 50 cells per condition). The data represent three independent experiments (**P<0.0001, ***P<0.01, unpaired Student's *t*-test). (E,F) PTP-PEST knockdown effects are blocked by p120 shRNA. (E) Stable knockdown of p120 was performed in HCT116 cells using retroviral infection with p120 or control shRNA, and confirmed by western blotting. (F) Co-expression of p120 shRNA and PTP-PEST siRNA reverses the effects of PTP-PEST knockdown on migration and Rho GTPase activity. Stable p120 shRNA or control shRNA HCT116 cells were transiently transfected with non-targeting siRNA or PTP-PEST smart pool siRNA to generate double-knockdown cells. Single knockdown of PTP-PEST (PTP-PEST siRNA) enhances HCT116 haptotaxis and Rac1 activity while suppressing RhoA. Co-expression of p120 shRNA blocks migration and Rho GTPase activity. **P<0.005 one-way ANOVA.

Cytosolic p120 has been reported to stimulate motility through modulation of Rho GTPase activity (Noren et al., 2000; Anastasiadis et al., 2000; Grosheva et al., 2001). Consistent with this, stable knockdown of PTP-PEST in HCT116 cells increased motility towards collagen I in a haptotaxis Transwell assay (Fig. 3A) and in KM12C cells as previously shown (Espejo et al., 2010). Since PTP-PEST and p120 are each known to reciprocally affect the activity of Rho GTPases (Noren et al., 2000; Sastry et al., 2006; Castaño et al., 2007; Yanagisawa et al., 2008; Espejo et al., 2010; Valls et al., 2012), we next assessed the activation of Rac1 and RhoA in HCT116-NT and HCT116-KD cells held in suspension or plated on collagen-I-coated dishes for 15, 30 and 60 minutes. Knockdown of PTP-PEST resulted in elevated Rac1 (Fig. 3B, top panel) and decreased RhoA activity (Fig. 3B, bottom panel) following attachment and spreading on collagen I when compared with control shRNA. This change in Rac1 and RhoA activity was accompanied by enhanced membrane ruffling and actin-rich protrusions in PTP-PEST knockdown cells (Fig. 3C,D). Strikingly, we observed enhanced distribution of p120 at the leading edge of lamellipodial protrusions following PTP-PEST knockdown when cells were spread on collagen. This leading edge distribution of p120 was further enhanced in the presence of EGF (Fig. 3C, arrows and 3D). To test whether p120 was required for the observed effects of PTP-PEST knockdown, we used shRNA to inhibit p120 expression. We generated a stable p120 shRNA knockdown in HCT116 cells along with a control shRNA cell line (Fig. 3E). We next transiently transfected PTP-PEST smart pool siRNA or non-targeting siRNA into these cell lines to generate double PTP-PEST- p120-deficient cells. Fig. 3F shows that PTP-PEST knockdown was unable to stimulate HCT116 haptotaxis in the presence of p120 shRNA. Similarly, p120 shRNA reversed the effect of PTP-PEST knockdown on Rho GTPase activity. Taken together, these results demonstrate that knockdown of PTP-PEST mimics the effects of p120 overexpression in epithelial cells and promotes its targeting to the leading edge in response to chemotactic signals. This further shows that the PTP-PEST knockdown effects are mediated by p120.

### PTP-PEST regulates the interaction of p120 with VAV2 and cortactin but not RhoA

**Fig. 4.**
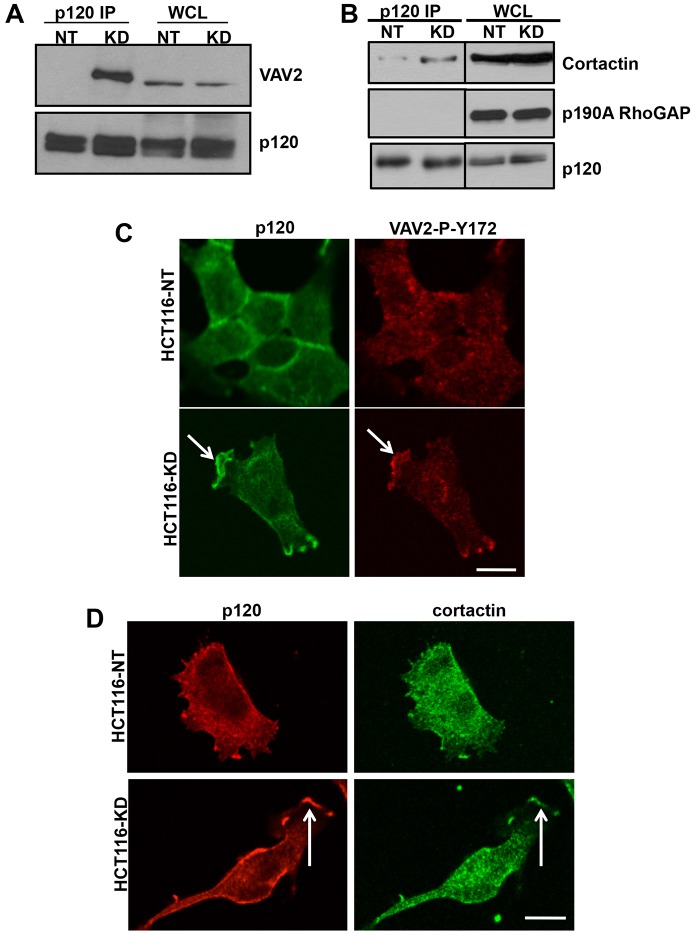
**PTP-PEST regulates interaction of p120 with VAV2 and cortactin.** (A) Endogenous p120 was immunoprecipitated from HCT116-NT and HCT116-KD cells. Western blot analysis with anti-VAV2 antibody shows an enhanced interaction of p120 with VAV2 in PTP-PEST knockdown cells. (B) Co-immunoprecipitation shows an increased association of endogenous p120 with cortactin but not p190 RhoGAP following PTP-PEST knockdown. (C) Immunofluorescence staining for p120 (green) and VAV2 (red), with anti-phospho-Y172 VAV2 (VAV2-P-Y172) shows that p120 and active VAV2 are localized at the leading edge in HCT116-KD cells (see arrows). (D) Immunofluorescence staining for p120 (red) and cortactin (green), shows that p120 and cortactin are localized at the leading edge in HCT116-KD cells (see arrows). In C and D, cells were plated on collagen-I-coated coverslips for 90 minutes followed by stimulation with EGF (25 ng/ml) for 15 minutes. Scale bars: 10 µm.

Several cytoplasmic binding partners of p120 have been identified that could mediate its effects on Rho GTPase activity and actin organization. These include the guanine exchange factor, VAV2 (Noren et al., 2000), cortactin (Boguslavsky et al., 2007), p190RhoGAP (Wildenberg et al., 2006) and RhoA (Anastasiadis et al., 2000; Castaño et al., 2007). We used immunoprecipitation to determine whether PTP-PEST affected the interaction of p120 with any of these proteins. We found that PTP-PEST knockdown promoted the interaction of p120 with VAV2 (Fig. 4A) and cortactin (Fig. 4B). Although both p190RhoGAP A and B isoforms were expressed in the cell types we tested, we were unable to detect an interaction of p120 with p190RhoGAP (Fig. 4B). Furthermore, we did not observe an interaction of p120 with RhoA (data not shown). The enhanced interaction with VAV2 and cortactin is consistent with increased localization of p120 at the leading edge of PTP-PEST knockdown cells. To confirm this, we determined if VAV2 and cortactin localized at the leading edge in PTP-PEST knockdown cells. As seen in Fig. 4C,D, active VAV2 (detected by anti-phospho-Y172 VAV2; Fig. 4C) and cortactin (Fig. 4D) were enriched, along with p120, at the leading edge of PTP-PEST knockdown cells following EGF stimulation. Thus PTP-PEST appears to affect interactions of p120 associated with membrane protrusion and lamellipodium formation, which could control localized Rac1 activity at the leading edge.

**Fig. 5.**
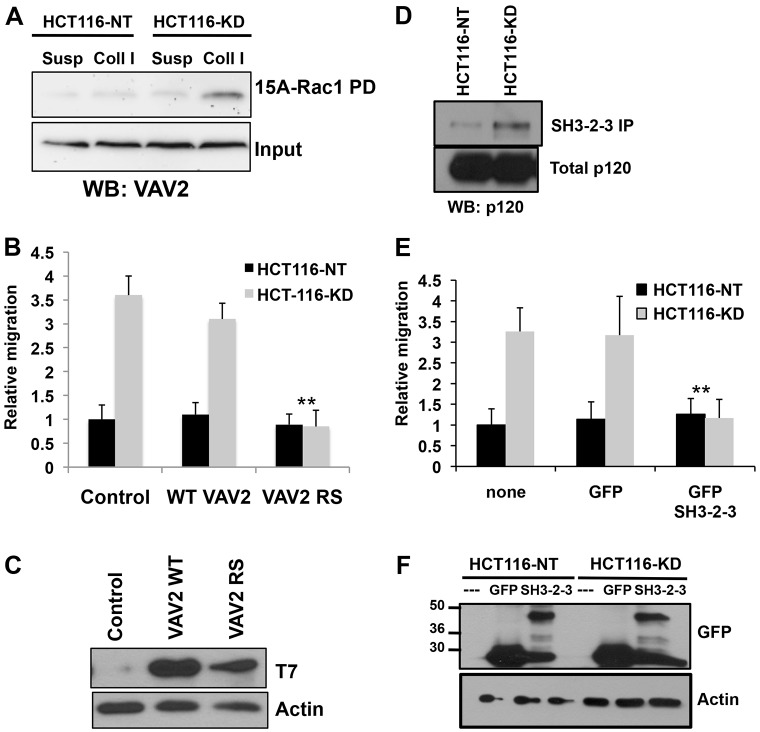
**VAV2 activity and interaction with p120 is required for enhanced motility.** (A) Nucleotide-free Rac1 pulldown assay in HCT116-NT and HCT116-KD cells in suspension (Susp) or plated on collagen I (Coll I) for 30 minutes shows increased VAV2 activity in PTP-PEST knockdown cells. (B) A DH domain mutant of VAV2, VAV2-RS, blocks enhanced motility in HCT-116-KD cells. **P<0.005, one-way ANOVA. (C) Western blot of T7 epitope tag shows equivalent levels of WT or mutant VAV2 expression. β-actin serves as a loading control. (D) A GFP-tagged C-terminal fragment of VAV2 encoding the SH3-SH2-SH3 domain shows increased interaction with endogenous p120 in HCT116-KD cells. GFP-SH3-SH2-SH3 cDNA was transiently transfected, immunoprecipitated with anti-GFP antibody (48 hours) and analyzed for p120 interaction by western blotting. (E) GFP–SH3-SH2-SH3 VAV2 expression blocks enhanced motility of HCT116-KD cells. **P<0.005, one-way ANOVA. (F) Western blot shows levels of GFP or GFP–SH3-SH2-SH3 VAV2 expression.

We next sought to test the functional significance of the enhanced p120–VAV2 interaction. Previous work from our lab showed that PTP-PEST regulates VAV2 GEF activity in fibroblasts (Sastry et al., 2006). To functionally link PTP-PEST and VAV2 in colon carcinoma cells, we measured VAV2 exchange activity in PTP-PEST knockdown cells using a nucleotide-free Rac1 (GST–15ARac1) pulldown assay (Sastry et al., 2006; García-Mata et al., 2006). HCT116-NT or HCT116-KD cells were either held in suspension or plated on collagen-I-coated dishes for 30 minutes. Fig. 5A shows that VAV2 interacted with GST–15ARac1 fusion protein in HCT116-KD cells but not in HCT116-NT cells. Using a dbl-homology (DH) domain point mutant of VAV2, L342R/L343S (VAV2-RS) (Marignani and Carpenter, 2001; Sastry et al., 2006), we next tested whether increased motility induced by PTP-PEST knockdown was dependent of VAV2 activity. When expressed in HCT116-KD cells, VAV2-RS, but not wild-type VAV2 was able to block haptotaxis towards collagen I (Fig. 5B,C). Previous studies showed that the effect of p120 overexpression could be blocked by co-expression of the C-terminal SH3-SH2-SH3 scaffolding domain of VAV2 (Noren et al., 2000). Therefore, we expressed this domain in PTP-PEST knockdown cells to test its effect on motility and p120 interaction. A GFP-tagged VAV2-SH3-SH2-SH3 construct was transfected into HCT116-NT or HCT116-KD cells. Immunoprecipitation with anti-GFP followed by p120 western blotting showed an enhanced interaction of p120 with the VAV2 C-terminal domain in HCT116-KD cells (Fig. 5D). Expression of the VAV2 C-terminal domain also blocked the migration of HCT116-KD cells (Fig. 5E,F). These results support a role for p120 and VAV2 in the enhanced motility of PTP-PEST knockdown cells.

### PTP-PEST targets a novel tyrosine site in the N domain of p120-catenin

**Fig. 6.**
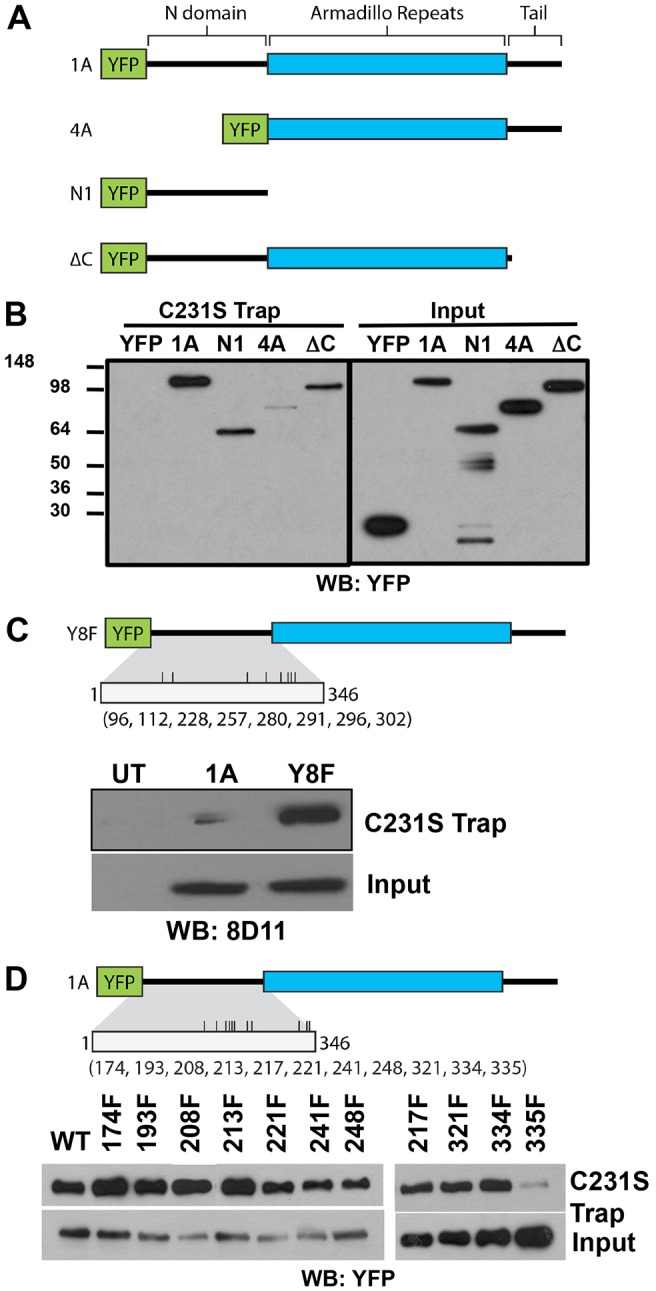
**PTP-PEST targets tyrosine 335 in the p120 N-terminal region.** (A) Schematics of the YFP-tagged deletion mutants of p120-1A that were tested for interaction with the PTP-PEST C231S trapping mutant. (B) GST–C231S traps p120-1A constructs containing the N-terminal domain. Full-length 1A isoform (1A), the N-terminal domain (N1) and the C-terminal deletion (ΔC) interact with the trapping mutant. The 4A isoform, lacking the N-terminal domain, does not interact with GST–C231S. (C) GST–C231S traps the Y8F p120-1A mutant showing that the eight indicated tyrosine residues are not target sites of PTP-PEST. (D) GST–C231S trapping of individual N-terminal tyrosine residue point mutants shows that Y335 is the PTP-PEST dephosphorylation site. Western blot with anti-YFP antibody used to detect trapped (top panels) versus input (bottom panels) p120 levels.

Several studies suggest that the 1A isoform of p120 stimulates motility and this activity is primarily attributed to the N-terminal region (Aho et al., 2002; Yanagisawa et al., 2008). Although the majority of known tyrosine sites in p120 are located in the N-terminal domain, several potential additional sites exist throughout the molecule (37 total sites). To determine the region of p120 targeted by PTP-PEST, we expressed YFP-tagged full-length p120 (YFP-1A), the N-terminal domain (YFP-N), the 4A isoform (YFP-4A) or a C-terminal deletion (YFP-ΔC) in HEK 293T cells and performed substrate trapping (Fig. 6A). Fig. 6B shows that YFP-1A, YFP-N1 and YFP-ΔC strongly interact with the trapping mutant whereas binding of the YFP-4A was less robust. The western blot of total protein expression shows comparable levels of expression for all constructs. This indicates that the tyrosine residue(s) targeted by PTP-PEST reside within the N-terminal domain of p120. This region is enriched in tyrosine residues and contains 19 potential phosphorylation sites (Fig. 6C,D). To narrow down the potential sites in this region, we expressed p120-Y8F, a mutant that abolishes eight of the nine known tyrosine phosphorylation sites (tyrosine residues 96, 112, 228, 257, 280, 291, 296, 302) (Mariner et al., 2001) and tested its ability to interact with the PTP-PEST-CS trapping mutant. As shown in Fig. 6C, the Y8F mutant retained its interaction with the PTP-PEST trapping mutant. Next, the 11 remaining tyrosine residues in the N-terminal domain were individually mutated to phenylalanine and tested for their ability to interact with the trapping mutant (Fig. 6D). This analysis resulted in the identification of tyrosine 335 as the site(s) of dephosphorylation by PTP-PEST. Fig. 6D shows that p120 Y335F is unable to bind to the C231S trapping mutant. In contrast, a point mutant of the neighboring tyrosine residue, Y334F, or a Y217F mutant (known to affect RhoA binding) (Castaño et al., 2007) were retained by the trapping assay pulldown.

**Fig. 7.**
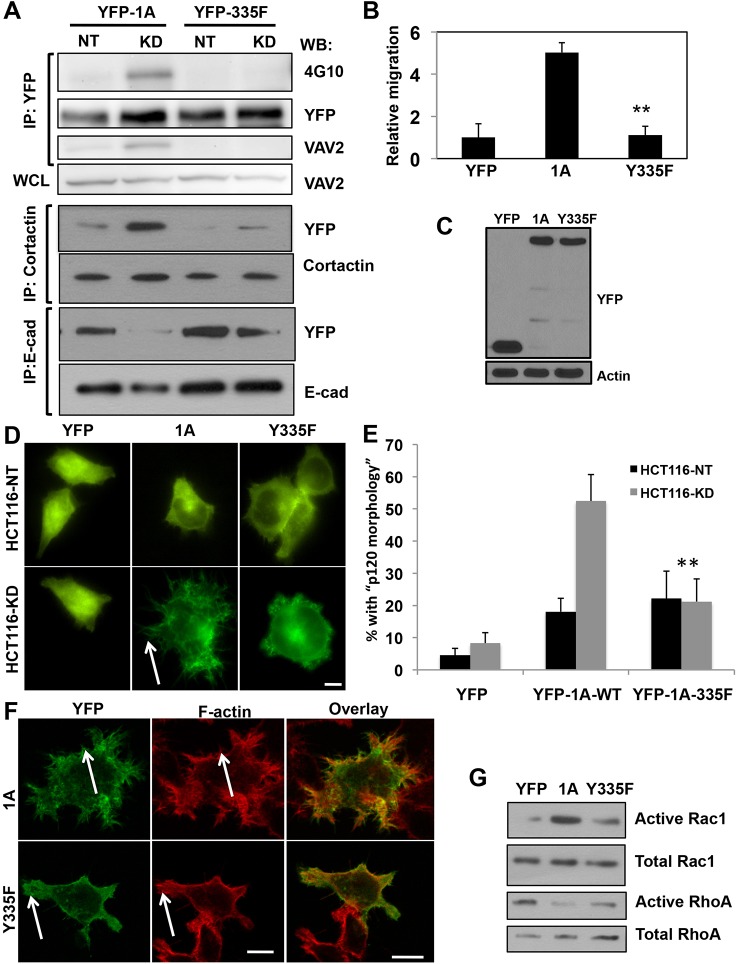
**Tyrosine 335 regulates epithelial migration and signaling to Rho GTPases.** Y335F mutant was tested for phenotypic and protein interaction effects. (A) Immunoprecipitation shows reduced tyrosine phosphorylation and decreased interaction of transiently expressed p120-1A-Y335F (YFP-335F) with VAV2 and cortactin and increased interaction with E-cadherin relative to wild-type p120-1A (YFP-1A) in HCT116-KD cells. (B,C) Transient expression of p120-Y335F does not stimulate motility relative to wild-type p120-1A or (D,E) induce a ‘p120 morphology’ when spread on collagen-I-coated coverslips for 1 hour. Scale bar: 10 µm. **P<0.005 one-way ANOVA. (F) YFP-1A (green) colocalizes with F-actin (red) in membrane protrusions (arrows) whereas YFP-Y335F shows a diffuse distribution and no colocalization with F-actin in HCT116-KD cells. Scale bars: 10 µm. (G) p120-Y335F suppresses Rac1 and activates RhoA relative to wild-type p120-1A in HCT116 cells plated on collagen-I-coated substrate for 1 hour.

To test the effect of the Y335F mutation on cell phenotype, this mutant was transfected into HCT116-NT or HCT116-KD cells and assayed for tyrosine phosphorylation by immunoprecipitation. In comparison with wild-type p120-1A, which is tyrosine phosphorylated in the HCT116-KD cells, the Y335F mutant showed a reduced level of phosphorylation (Fig. 7A). This mutant also showed reduced association with VAV2 and cortactin and an increased interaction with E-cadherin when expressed in HCT116-KD cells (Fig. 7A). In agreement with previous studies (Noren et al., 2000; Anastasiadis et al., 2000; Grosheva et al., 2001; Soto et al., 2008), overexpression of wild-type p120-1A in parental HCT116 cells enhanced migration in a haptotaxis assay towards collagen I (Fig. 7B,C). In contrast, overexpression of the Y335F mutant had no effect on cell motility. An immunofluorescence assay showed that YFP–p120-1A wild type induced more prominent membrane protrusions and filopodia in HCT116-KD relative to HCT116-NT cells (Fig. 7D,E) and colocalized with F-actin in protrusions (Fig. 7F). In contrast, the Y335F mutant did not significantly alter cell morphology or localize in actin-rich protrusions (Fig. 7D–F). Finally, whereas overexpression of wild-type p120 enhanced Rac1 and decreased RhoA activity in HCT116 cells, expression of the Y335F mutant did not stimulate Rac1 activity and had a modest effect on RhoA (Fig. 7G).

## DISCUSSION

**Fig. 8.**
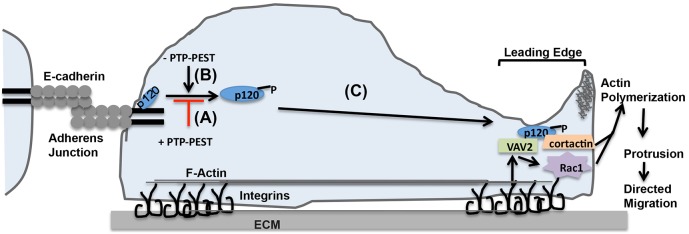
**Model for the role of PTP-PEST in cell–cell junctions versus leading edge protrusion.** (A) PTP-PEST localizes at adherens junctions to promote E-cadherin–p120 interaction, thereby stabilizing adherens junctions. (B) Inactivation of PTP-PEST enhances tyrosine phosphorylation of Y335 and displaces p120-Y335-*P* to the cytosol. (C) p120-Y335-*P* localizes to the leading edge where it interacts with VAV2 and cortactin to promote Rac1 activity and actin polymerization leading to enhanced protrusion and directed migration.

In this study, we identified p120 catenin as a substrate of the protein tyrosine phosphatase, PTP-PEST, in epithelial cells. Our data demonstrate that PTP-PEST dephosphorylates a previously unidentified site, Y335, in the N-terminal regulatory domain of p120. We further show that PTP-PEST controls the shuttling of p120 from an E-cadherin-bound to a cytosolic pool. Silencing of PTP-PEST results in increased cytosolic p120, increased motility, and altered Rho GTPase activity through interaction with VAV2. The enhanced localization of p120 at lamellipodia in PTP-PEST knockdown cells suggests that PTP-PEST controls the recruitment and/or activation of Rac1 signaling complexes at the leading edge (see model in Fig. 8). Taken together, our data suggest that dephosphorylation of p120 by PTP-PEST may suppress epithelial motility by affecting the subcellular distribution of p120 and its availability to regulate localized Rho GTPase activity.

It is well established that p120 promotes both static epithelial cell–cell adhesion and motility (Reynolds and Roczniak-Ferguson, 2004; Anastasiadis, 2007). These dual effects can be attributed in part to the subcellular localization of p120. To date, few studies have dissected the regulatory pathways controlling p120 association with E-cadherin versus its cytoplasmic localization. The phosphorylation of p120, either on tyrosine or serine/threonine, has been proposed to control its interaction with E-cadherin to affect adhesive function (Fukumoto et al., 2008; Petrova et al., 2012). In support of this, a recent study showed that phosphorylation of serine residues in the p120 N-terminal domain affects the E-cadherin activation state (Petrova et al., 2012). The role of tyrosine residues in this process has not been fully addressed. To date, all known tyrosine phosphorylation sites, as well as the N-terminal domain itself, have been shown as dispensable for binding to E-cadherin (Castaño et al., 2007; Macpherson et al., 2007), although the effect of these sites on E-cadherin activation have not been investigated. Although p120 lacking the N-terminal domain (4A isoform) is sufficient to bind to E-cadherin, recent structural studies have shed light on a complex binding interface between p120 and the E-cadherin juxtamembrane domain (JMD) in which the JMD contains both dynamic and static binding sites for p120 (Ishiyama et al., 2010). How the N-terminal domain affects this binding interface has not been examined. Computational analysis of the p120 N-terminal domain indicates it is largely unstructured (Orlichenko et al., 2010). Thus it is conceivable that phosphorylation of the N-terminal domain could modulate p120 conformation and its association with E-cadherin JMD sites or other proteins. For example, tyrosine phosphorylation of residues 112 and 228 creates a binding site for RhoA in the N-terminal domain and together with a RhoA-binding site in the armadillo domain, may cause p120 to adopt a folded structure in which the N-terminal domain and armadillo domain are bridged by RhoA (Anastasiadis et al., 2000; Yanagisawa et al., 2008; Castaño et al., 2007). Our results presented here indicate that tyrosine 335, which is located proximal to the interface between the N-terminal domain and the armadillo region (residue 347), is a novel site of phosphorylation. Although the structural role of this site relative to the E-cadherin-binding and armadillo domains is not known, it is conceivable that the phosphorylation of Y335 on p120 could alter its conformation or interaction with a cytoplasmic protein, precluding its interaction with E-cadherin. Alternatively, changes in E-cadherin conformation, due to disruption of Rac1 and RhoA activity in response to PTP-PEST knockdown, may alter the accessibility of p120 to its juxtamembrane binding sites on E-cadherin. Each of these scenarios would favor the enhanced distribution of p120 in the cytosol. Our results showing reduced association of p120 with E-cadherin along with the increased levels in the cytoplasm in the absence of PTP-PEST support this view. It remains to be seen how this reduced binding of p120 to E-cadherin affects junctional stability; however, we did not observe any gross alterations in E-cadherin cell surface expression under steady state conditions in NT or KD cells relative to parental cells (Espejo et al., 2010). Likewise, we did not observe any changes in total expression levels of p120 or other catenins (Espejo et al., 2010). Finally, knockdown of PTP-PEST did not induce degradation of p120 (Y.J., unpublished observations), suggesting that PTP-PEST regulates the distribution of p120, but not its expression or stability. As we have previously shown that PTP-PEST is required for junctional assembly, it will be important to determine whether this is due to a direct effect of p120 on E-cadherin or through an indirect effect of PTP-PEST on Rho GTPases or another downstream target.

Several studies have shown that overexpression of p120, which enhances the cytoplasmic pool, results in enhanced motility, increased Rac1 and decreased RhoA activity. The phenotype of PTP-PEST KD cells closely resembles the previously observed effects of p120 overexpression. We observed enhanced cytosolic p120 along with elevated Rac1 and reduced RhoA activity in KD cells plated on collagen substrates. We also found that p120 is enhanced in lamellipodia in PTP-PEST KD cells. Therefore, one role of PTP-PEST may be to restrict the localization of p120 from the leading edge. p120 was reported to colocalize with cortactin at the leading edge where it promotes actin polymerization and where it may serve to localize Rac1 activity (Boguslavsky et al., 2007). How p120 localizes Rac1 activity at the leading edge is not known. The enhanced interaction of p120 with VAV2 and cortactin that we observe suggests a potential mechanism. Our data support a model in which p120 recruits a complex consisting of VAV2 and cortactin to the leading edge to increase Rac1 activity and stimulate actin polymerization to drive membrane protrusion (Fig. 8). Our data demonstrate that PTP-PEST regulates phosphorylation of Y335 in the N-terminal domain of p120, and this phosphorylation plays an important role in the interaction of p120 with VAV2 and activation of Rac1. This suggests that p120, when phosphorylated on Y335, interacts with VAV2, probably through its SH2 domain to activate Rac1 at the leading edge to promote membrane protrusion and motility. In support of this, we observed that overexpression of the Y335F mutant did not lead to increased motility relative to wild-type p120-1A. Thus, our data support a role for PTP-PEST in regulating p120–VAV2 interactions leading to activation of Rac1 and subsequent actin polymerization mediated by cortactin. Surprisingly, we found that PTP-PEST knockdown suppressed RhoA activation; however, we found no evidence for PTP-PEST controlling the binding of p120 to RhoA. Thus, it is possible that PTP-PEST suppresses RhoA as a downstream consequence of elevated Rac1 activity (Nimnual et al., 2003; Wildenberg et al., 2006) or through an alternate mechanism.

Previous studies have suggested that the cytosolic distribution and targeting of p120 to the leading edge play a crucial role in epithelial cell motility and tumor cell invasion (Noren et al., 2000; Anastasiadis et al., 2000; Grosheva et al., 2001, Boguslavsky et al., 2007). However, the mechanisms controlling this activity have been largely undetermined. Our previous studies, along with those of others, demonstrate that PTP-PEST regulates motility of numerous epithelial cancer cell types (Espejo et al., 2010; Mathew et al., 2008; Taieb et al., 2008; Zheng et al., 2009; Sun et al., 2011). Here, we provide evidence that PTP-PEST mediates these effects through regulation of a distinct tyrosine site (Y335) in the p120 N-terminal domain to control p120 subcellular distribution. Interestingly, the effects of PTP-PEST on p120 phosphorylation appear to be specific to epithelial cells. We did not observe enhanced phosphorylation of p120 in PTP-PEST null fibroblasts (S.S., unpublished observations) or in endothelial cells treated with PTP-PEST siRNA (K. Burckart, personal communication). This suggests that an epithelial-specific kinase may be responsible for the enhanced phosphorylation. Our findings could have significant implications for epithelial tumor cell invasion and metastatic progression. In particular, increased cytosolic localization of p120 is associated with highly aggressive, metastatic disease in colon cancer (Bellovin et al., 2005) and with anoikis resistance in lobular breast cancer (Schackmann et al., 2011). Thus, future studies addressing which kinases phosphorylate p120 and how downstream signals drive migration, invasion and survival will provide valuable insight and novel avenues for therapeutic intervention.

## MATERIALS AND METHODS

### Cell lines, antibodies and reagents

KM12C colon carcinoma cells (Morikawa et al., 1988) were cultured in MEM (Cellgro) supplemented with 10% fetal bovine serum (FBS; Cellgro), non-essential amino acids, MEM vitamin solution and sodium pyruvate, as described previously (Espejo et al., 2010). HCT116 colon carcinoma cells were maintained in McCoy's 5A (Cellgro) medium supplemented with 10% FBS. HEK 293T cells were maintained in DMEM (Sigma) containing 10% FBS. Epidermal growth factor was purchased from Sigma. Rat-tail collagen type I was purchased from BD Biosciences. Monoclonal antibodies to p120, E-cadherin, α-catenin, p190 Rho GAP, cortactin, Living Colors™ anti-YFP antibody, JL-8, and Rac1 were purchased from BD Biosciences. Anti-E-cadherin antibody, DECMA-1, was purchased from Abcam. Mouse-specific p120 antibody, 8D11, was a generous gift from A. Reynolds (Vanderbilt University, Nashville, TN). Polyclonal rabbit anti-p120 antibody was obtained from Santa Cruz and rabbit antiserum to β-catenin was purchased from Sigma. Rabbit anti-PTPN12 (PTP-PEST) was purchased from Sigma. Anti-phosphotyrosine antibody, 4G10, was purchased from Millipore. VAV2 antibodies were used as described previously (Liu and Burridge, 2000) or purchased from Cell Signaling. Anti-phospho-Y172-VAV2 antibody was purchased from Abcam. RhoA antibody was purchased from Santa Cruz.

### Plasmid vectors and site directed mutagenesis

HA-tagged wild-type p120-1A and p120-Y8F constructs (Mo and Reynolds, 1996) and YFP-tagged p1201A, p1204A and p120N1 constructs (Soto et al., 2008; Yanagisawa et al., 2008) were generated as described previously. The GFP-tagged C-terminal deletion of p120 (p120-ΔC) was a gift from A. Nagafuchi (Kumamoto University, Japan) (Liu et al., 2007). Mutation of tyrosine sites in p120 were made using the QuikChange II Site-Directed Mutagenesis Kit™ from Stratagene (Santa Clara, CA) following the recommended protocol. The following primers were utilized: Y174F 5′-agcagtctccaataactttatccagaccttgggcc-3′ and 5′-ggcccaaggtctggataaagttattggagactgct-3′; Y193F 5′-gggccctggtccctttgtggggc-3′ and 5′-gccccacaaagggaccagggccc-3′; Y208F 5′-cactcttcctaggaacttccactttcctccagatgg-3′ and 5′-ccatctggaggaaagtggaagttcctaggaagagtg-3′; Y213F 5′-cactatcctccagatgggtttggccgacac-3′ and 5′-gtgtcggccaaacccatctggaggatagtg-3′; Y221F 5′-gccgacactatgaagatggttttccaggtggca-3′ and 5′-tgccacctggaaaaccatcttcatagtgtcggc-3′; Y241F 5′-gaattgaggagcggtttaggcccagcatgga-3′ and 5′-tccatgctgggcctaaaccgctcctcaattc-3′; Y248F 5′-gcccagcatggaaggcttccgggcacc-3′ and 5′-ggtgcccggaagccttccatgctgggc-3′; Y217F 5′-cagatgggtatggccgacactttgaagatggttatc-3′ and 5′-gataaccatcttcaaagtgtcggccatacccatctg-3′; Y321F 5′-gacgacgcctcaggagctttgaagacatgattgg-3′ and 5′-ccaatcatgtcttcaaagctcctgaggcgtcgtc-3′; Y334F 5′-gtgccgcctgatcagttctattgggctccttta-3′ and 5′-taaaggagcccaatagaactgatcaggcggcac-3′; Y335F 5′-ccgcctgatcagtacttttgggctcctttagct-3′ and 5′-agctaaaggagcccaaaagtactgatcaggcgg-3′. All mutated sequences were confirmed by DNA sequencing using primer 5′-aggagccaggacagattgtggaaa-3′. VAV2 mammalian expression constructs (T7-VAV2 wild type and T7-VAV2-RS) and GFP-VAV2-SH3-SH2-SH3 were previously described (Noren et al., 2000; Marignani and Carpenter, 2001; Sastry et al., 2006). Plasmids were transiently transfected into cell lines as indicated using Lipofectamine Plus reagent (Invitrogen) as described previously (Espejo et al., 2010).

### PTP-PEST and p120 knockdown

Smart pool human PTP-PEST-specific siRNA or non-targeting control siRNA was purchased from Thermo-Scientific and introduced by electroporation as described previously (Espejo et al., 2010). Knockdown of PTP-PEST was verified by western blotting after 48 hours. For stable shRNA knockdown, KM12C or HCT116 cells were infected with retroviruses (pSUPER-retro-puro) expressing PTP-PEST-specific shRNA or a scrambled control shRNA and selected with 1 µg/ml puromycin (Espejo et al., 2010). Puromycin-resistant cells were pooled and used as a polyclonal pool for all experiments. Resulting cell lines are referred to as KM12C-NT, KM12C-KD, HCT116-NT and HCT116-KD. For stable knockdown of p120 in HCT116 cells, control or p120 shRNA was expressed using retroviral infection (pSUPER-retro-puro) followed by puromycin selection as described (Yanagisawa and Anastasiadis, 2006). Knockdowns were verified by western blot analysis for PTP-PEST or p120.

### Cell fractionation

Cells were collected from culture dishes using a rubber policeman on ice and pelleted at 1000 rpm for 5 minutes. Cells were resuspended in four times the packed cell volume of hypotonic buffer (10 mM Tris-HCl, pH 7.4, 1 mM MgCl_2_, 1 mM EDTA, 1.5 mM PMSF, 0.5 mM NaVO_4_, 10 mM NaF, 10 µg/ml aprotinin, 10 µg/ml leupeptin) and allowed to swell on ice for 10 minutes. Cells were lysed in a dounce homogenizer and the final NaCl concentration was adjusted to 150 mM. Nuclei were pelleted by centrifugation (3300 ***g***, 15 minutes) and the resulting supernatant was centrifuged at 100,000 ***g*** for 30 minutes to obtain cytosolic (supernatant) and membrane (pellet) fractions. Protein concentrations were adjusted to 1 mg/ml. Nuclei were resuspended in 20 mM Hepes, pH 7.9, 0.4 M NaCl, 1 mM EDTA, 1 mM EGTA, 1 mM DTT, 10% glycerol, 1.5 mM PMSF, 0.5 mM NaVO_4_, 10 mM NaF, 10 µg/ml aprotinin, 10 µg/ml leupeptin), incubated on ice for 30 minutes, then centrifuged at 12,000 ***g*** for 5 minutes to pellet large debris and nucleic acid. 

### Western blots and immunoprecipitation

E-cadherin immunoprecipitations were performed using DECMA-1 monoclonal antibody. HCT116-NT or HCT116-KD cells were grown to subconfluency, washed with PBS on ice and DECMA-1 or murine IgG (10 µg/ml in D-PBS + calcium and magnesium) was bound to the cells for 1 hour on ice. Excess antibody was washed with PBS and cells were lysed in 50 mM Tris pH 7.6, 150 mM NaCl, 0.5% NP-40 + protease inhibitors (10 µg/ml aprotinin, 10 µg/ml leupeptin, 1 mM PMSF). Endogenous p120 was immunoprecipitated with monoclonal p120 antibody (BD Biosciences) from cells lysed in modified RIPA buffer containing protease inhibitors [10 µg/ml aprotinin, 10 µg/ml leupeptin, 1 mM phenylmethylsulfonyl fluoride (PMSF)], 100 mM sodium orthovanadate and 10 mM sodium fluoride. Transfected YFP-tagged p120 constructs were immunoprecipitated in the same lysis buffer using Living Colors™ monoclonal antibody, JL-8. Lysates were centrifuged for 10 minutes at 13,200 rpm at 4°C. For all immunoprecipitations, cleared lysates were incubated with 25 µl of protein-A–Sepharose beads for 1–2 hours to collect antibody–antigen complexes. Beads were washed several times with lysis buffer and bound proteins released by boiling in Laemmli sample buffer and separated by SDS-PAGE (Laemmli, 1970). E-cadherin immunoprecipitates were probed by western blot analysis (Towbin et al., 1979) for p120, α-catenin, β-catenin and E-cadherin. p120 or YFP–p120 immunoprecipitates were probed for 4G10, YFP, p190 RhoGAP, cortactin or VAV2 as indicated in the figure legends. Where indicated, β-actin was used as a loading control.

### Substrate trapping

Substrate trapping and GST fusion protein isolation was performed as described previously (Flint et al., 1997; Sastry et al., 2006). Briefly, endogenous or transiently transfected proteins were ‘trapped’ by treating cells with pervanadate (50 µM, 20 minutes) followed by cell lysis on ice in modified RIPA buffer (50 mM Tris pH 7.6; 150 mM NaCl, 1% Triton X-100, 0.25% sodium deoxycholate, 2 mM EGTA) plus 10 µg/ml aprotinin, 10 µg/ml leupeptin, 1 mM PMSF. For 1 mg of cell lysate, 10 µg of GST, GST–PTP-PEST-WT or GST–PTP-PEST-C231S fusion protein was added and incubated with rotation at 4°C for 1 hour. Trapping pulldowns were washed in lysis buffer and beads were boiled in Laemmli sample buffer. Trapped proteins were detected by western blot analysis.

### GTPase and GEF activity assays

To measure Rac1 and RhoA activity in PTP-PEST knockdown cell lines, cells stably expressing non-targeting or PTP-PEST shRNA were trypsinized, washed in serum-free medium containing 1% BSA and then either held in suspension or plated on collagen-I-coated dishes for the indicated times. To measure Rac1 and RhoA activity for p120 mutants, HCT116-NT or HCT116-KD cells were transiently transfected with YFP, YFP–p120-1A or YFP–p120-1AY335F as described above. Cells were held in suspension or plated on collagen-I-coated dishes prior to lysis. Rac1 and RhoA activity assays using GST–PBD or GST–Rhotekin–RBD fusion proteins were performed as described previously (Sastry et al., 2006). p120 binding to N19-RhoA was performed by incubating 10 µg of GST–N19-RhoA (gift of K. Burridge, UNC-Chapel Hill, NC) or GST only with the indicated lysates for 1 hour (Yanagisawa et al., 2008). VAV2 exchange factor activity was measured using a nucleotide-free Rac1 pulldown assay with a GST–15ARac1 fusion protein in HCT116-NT versus HCT116-KD cells held in suspension or plated on collagen I-coated dishes as described previously (Sastry et al., 2006; García-Mata et al., 2006).

### Cell migration assays and immunofluorescence

All cell migration assays were performed using 8 µm Transwell inserts (Costar) as described previously (Espejo et al., 2010). Haptotaxis was tested by coating the bottom membrane of the Transwell insert with collagen I (15 µg/ml in PBS). 50,000 cells were added to the upper chamber in serum-free medium containing 1% BSA and cells were allowed to migrate for 4 hours at 37°C in a 5% CO_2_ incubator. The assays were terminated by fixation in 3.7% formaldehyde for 10 minutes. Non-migrated cells were removed with a cotton swab and the lower membrane was stained with 4′,6-diamidino-2-phenylindole (DAPI, 5 ng/ml) for 30 minutes. Migration was scored as the number of nuclei per high-power field for five random fields using an inverted Nikon TE2000 epifluorescence microscope equipped with a UV excitation/emission filter. Immunofluorescent staining of p120 was performed by plating control (NT)- or shRNA (KD)-expressing cell lines on collagen-coated glass coverslips for 1 hour in serum-free medium containing 1% BSA followed by treatment with 25 ng/ml EGF for 15 minutes. Cells were fixed in 3.7% paraformaldehyde in PBS, permeabilized in 0.5% Triton X-100 in PBS and washed with PBS. Coverslips were blocked in 10% goat serum in PBS for 1 hour at room temperature. Cells were stained for endogenous p120 (BD Biosciences), cortactin, phospho-Y172–VAV2 and F-actin. Alexa-Fluor-488- or -594-conjugated secondary antibodies and phalloidin were obtained from Molecular Probes (Invitrogen). Primary and secondary antibodies were diluted in 10% goat serum in PBS. Images were obtained using a Zeiss LSM 510 confocal microscope with a 1.4 NA, 63× oil-immersion objective. Optical slices were 1 µm thickness. The percentage of cells with lammellipodia and p120 leading edge localization was determined for five random fields (50–100 cells). To assess morphology of p120 mutants, transiently transfected cells were plated on collagen-coated coverslips for 1 hour, fixed and stained for actin with Alexa-Fluor-594–phalloidin. The percentage of YFP-positive cells with ‘p120’ morphology was determined for five random fields (50–100 cells). All assays were performed in triplicate in at least three independent experiments. Statistical significance was determined by Student's *t*-test or one-way analysis of variance (ANOVA) analysis using Graphpad Prism software.
